# A pedagogical approach to science outreach

**DOI:** 10.1371/journal.pbio.3000650

**Published:** 2020-04-16

**Authors:** Marni B. McClure, Kacey C. Hall, Erin F. Brooks, Catherine T. Allen, Kenneth S. Lyle

**Affiliations:** 1 Department of Chemistry, Duke University, Durham, North Carolina, United States of America; 2 Lineberger Comprehensive Cancer Center, University of North Carolina, Chapel Hill, North Carolina, United States of America

## Abstract

Encouragement of students across all communities through scientific outreach programs is critical to engaging the next generation, exciting young minds to pursue careers in science and medicine. Herein, we present a uniquely structured and widely influential science outreach program. Founded in 2005, the Duke Chemistry Outreach (DCO) employs a pedagogical approach to outreach that aims to teach its audience a new scientific concept, while instilling a pure enjoyment of science. DCO has performed 583 events reaching over 70,000 participants throughout 2,270 hours, with the majority of events in Durham, the surrounding North Carolinian communities, and across 8 other states. The flexibility and diversity of this outreach program creates a framework amendable for others to adopt in both secondary and higher education settings.

## Abstract 2

Across 14 years, 581 events, and reaching 70,000 audience members, the Duke Chemistry Outreach program has engaged the surrounding community through fun scientific demonstrations. This Community Page article provides examples and guidelines to encourage others to establish similar programs.

## Introduction

Science outreach is a vital connection between the scientific community and the general public [1–3]. Outreach encourages youths who may be otherwise disconnected from pursuing science, engineering, technology, and math (STEM)–related fields in which there remains a paucity of minorities [4]. Furthermore, it teaches those presenting effective science communication skills, a desperately needed skill in the current climate of fake news and scientific misinformation [5,6]. Finally, universities have an obligation to share the knowledge, education, and love of learning with their surrounding communities [7].

Duke Chemistry Outreach (DCO) provides a promising model for widespread community impact through an undergraduate and graduate volunteer program. Through 14 years of outreach within the Durham area, across North Carolina, and the greater Eastern seaboard, DCO presented 581 events, conducted about 2,270 hours of outreach, and has had close to 70,000 audience members (S1 Table). The impact of this program on the undergraduate and graduate students who participated is far-reaching. Herein, we describe the establishment of our program, outcomes of the students participating, and resources for the community in an effort to provide a roadmap for the establishment of similar programs.

## Outreach: Sharing the joy of science

DCO utilizes volunteers to perform chemistry demonstrations with the goal of instilling a joy of science. Prior to each event, the DCO coordinator personally meets with each community partner that has requested a performance in person to see the space for presentation, understand any limitations (i.e., can open flame be used and how to best engage the audience with the given space), and identify the goal for the presentation prior to organizing the program. For example, some outreach events included a review of a chemistry topic the students had been learning in science class. Other community partners desired a holiday theme such as Halloween’s “Chem-Mystery” Night, Science Under the Stars, and liquid nitrogen ice cream. Eliciting the goal of the presentation from the community partner is critical for effective informal chemistry learning [8] and allowed the DCO coordinator to incorporate multiple exciting and engaging demonstrations that focus on a single scientific concept. Importantly, even if the same demonstrations can be used to illustrate more than one concept, only one concept is highlighted per event.

The goal of DCO is for the learner to gain a deeper understanding of the concept through effective communication and to foster a love for science. Favorite demonstrations include the following: (1) filling soap balloons with gasses of varying densities and observing how the balloons sink or rise and then lighting propane-filled bubbles to demonstrate that propane is both denser than air and flammable; (2) producing polyurethane foam smoothies that warm due to the exothermic reaction; and (3) demonstrating acid/base properties by blowing bubbles through water with universal indicator (UI), dropping dry ice into water with UI, or putting antacids such as milk of magnesia into slightly acidic water with UI and watching the color change (for a more complete list of demonstrations, see S2 Table). Performing activities such as these not only assists children with a better understanding of chemical concepts but also creates an enthusiasm for science. The audience almost always leave with big smiles, joyfully talking about what they did or saw.

## Community partners

**Fig 1:**
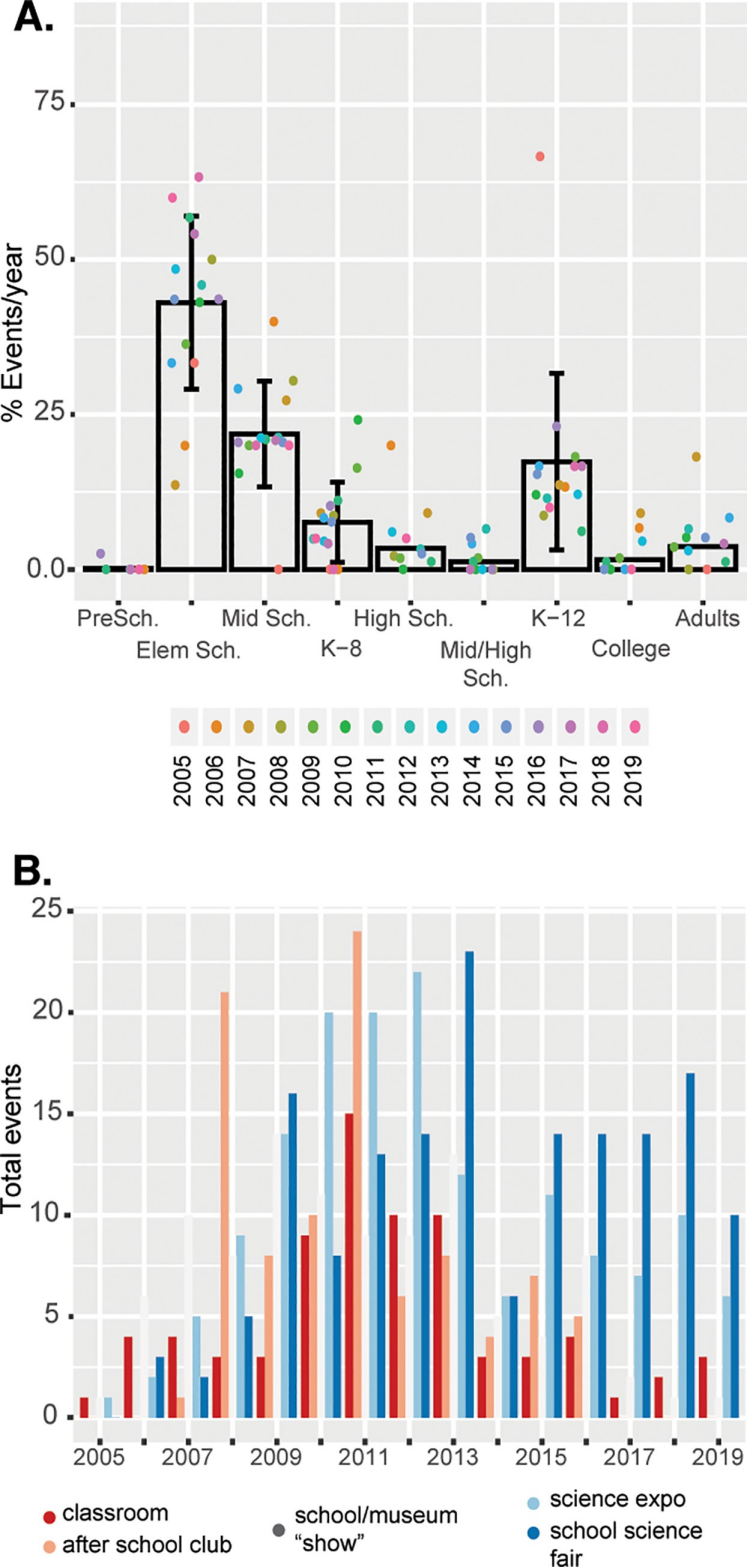
Distribution of events by audience and venue type. DCO has performed 580 events to date. A. Distribution of events per year, with each dot colored by year. B. Total number of events per year at various outreach event types, including school classrooms, science fairs, after-school camps, science conferences, large stage shows for the general public, and at local museums (S1 Table). DCO, Duke Chemistry Outreach; Elem, elementary; Mid, middle; PreSch, preschool; Sch, school.

The first outreach events began in collaboration with Duke University Department of Chemistry instructor Dr. Kenneth Lyle engaging local teachers in the community with chemistry demonstrations for their classrooms. A colleague invited Dr. Lyle to perform an outreach event at the local Museum of Life and Science. As visibility began to increase through community partner requests, connections with other educators led to events at elementary schools with regular science fairs, the NC State Fair, and events at the Museum (Fig 1, S1 Table). Teachers and parents from prekindergarten/elementary through high schools began requesting DCO events, leading to involvement in-school, after-school and summer science clubs both on and off Duke University’s campus, large stage shows during the Duke Alumni Weekend, and a fall show of Science Under the Stars. All events are free. Elementary school outreach is the most common event compared to middle and high school outreach (Fig 1A, ANOVA *p*-value: 8 × 10^−8^).

**Fig 2:**
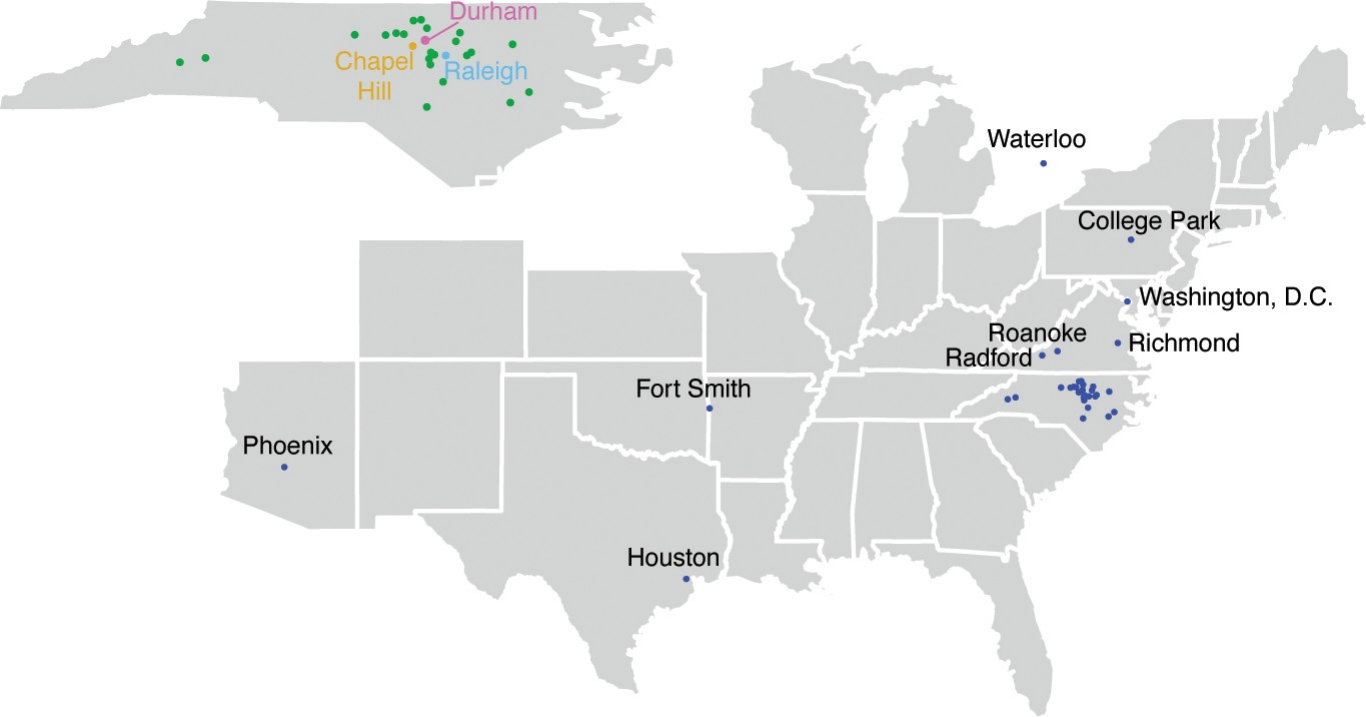
Locations of DCO events. Concentrated in the Durham and surrounding Research Triangle, DCO participated in events across North Carolina, Virginia, with additional events in Arizona, Texas, Pennsylvania, and Canada (S1 Table). DCO, Duke Chemistry Outreach.

Initially, events were concentrated around Duke University and the Durham area, with 409 events total. With time, our program expanded throughout the surrounding Research Triangle area, with 74 events in Raleigh and 11 events in Chapel Hill. DCO participated in science days and classroom presentations across 7 other states (Fig 2, S1 Table, created using ggplot2 [9] ggmap [10]).

The growth of DCO towards more school science fairs and stage shows is a result of community partner demand. Certainly, after-school programs are extremely time demanding and require a consistent group of students committed to volunteering each week. This is compared to a science night, which could be a once-a-semester or even once-a-year activity. Given the involved nature of putting together our presentations, the science nights and exposés became more common.

## Presenters

Volunteers for DCO were initially Duke University undergraduate and graduate students within the Department of Chemistry; however, with the initiation of a service-learning course enrolling 15–20 students per semester across all departments in 2008, the program grew substantially both in event number and participants. Professors, members of the instructional staff, office staff, and postdoctoral fellows volunteered their time. As outreach events broadened across the Raleigh–Durham–Chapel Hill area, volunteers from North Carolina State University, the University of North Carolina, the Museum of Life Science, industry partners, high school students, teachers, and parents all graciously volunteered as presenters. Other large organizations became involved as well as DCO grew, including the Duke Lemur Center, Departments of Physics, Biology, Engineering, and Molecular Genomics and Microbiology, and the Duke Strategic Materials Imaging Facility. These groups contributed both demonstrations and actively volunteered in DCO.

The most significant requirement for the volunteers is a commitment to understanding the presentation, communicate effectively through practice, and share the love of science with the surrounding community. Volunteers were required to practice the upcoming outreach event at least once in the laboratory, and often more than once, to be comfortable with a new set of demonstrations. In this practice session, volunteers received feedback from the program director. Students in the service-learning course received significant feedback and training.

## Chemistry Outreach Service Learning course

In the fall of 2008, the program coordinator along with one teaching assistant established the Chemistry Outreach Service Learning course. As a cross-listed course in the Department of Service Learning, this course required students to perform over 20 hours of outreach per semester. Through this course, we required each student to practice an outreach event in front of the class, while also being video recorded. The student then received immediate feedback from his/her peers and the program coordinator as well as performing self-reflection after watching the video [11]. We provided reflection pieces on the importance of community outreach, communication tools, and the public policy questions that can arise from such programs. Our program allowed the DCO program, as a whole, to grow substantially, with a significant infusion of excited undergraduate students invested in education and volunteering. We have included our syllabus and reading material topics to help others establish and grow a similar service-learning course (S1 Text, S3 Table).

## Student outcomes

### Ethics statement

The Duke University Institutional Review Board (IRB) for Non-Medical Research approved this research Federal Wide Assurance number 265 by written consent.

While direct measurements on participants were not undertaken, 2 semesters of the Chemistry Outreach Service Learning course were subjected to an IRB-approved study to examine the impact of DCO on Duke University undergraduates. In total, 35/35 students concurred that DCO is a very positive experience, with the majority citing growth in presentation skills, self-confidence, and understanding ways to keep an audience engaged. One student recollected that after hearing children state “I want to go to college to do this” truly understood that her outreach experience was inspiring the next generation. Furthermore, students stated that they felt they “can really make a difference” and cited DCO as a reason for pursuing teaching as a profession. Significant challenges in the course were the time commitment, but all but one student stated that they wished to continue volunteering. Four former students from the Chemistry Outreach Service Learning course have not only become science educators but also opened their own science outreach programs.

## Keys to Success

Fourteen years of continued and successful outreach through DCO has brought an understanding of what is needed to create an effective program outline. Here, we offer 4 points that we consider to be invaluable to the advancement of similar programs:

1. **Energetic and passionate leader for the program.** An enthusiastic leader is vital to any program that aims to ignite interest, excitement, and curiosity in the field of science. A single point person must be willing to reach out to and engage with teachers and new venues. Once a contact is made, personal partnership with the community partner is essential so that each outreach event can be tailored specifically for the target audience. To achieve this, the DCO program coordinator personally met with each new community partner at the location of outreach prior to designing the event. DCO relied heavily on its program director for the initial success and continued expansion of the program.
  Duke University provided a unique and special environment to allow for the creation of such a program. By hiring Dr. Lyle to perform experiments and demonstrations for the Department of Chemistry, the department provided both protected time and resources for the establishment and growth of DCO. Dr. Lyle’s previous experience performing chemistry outreach, coupled with this support, allowed DCO to flourish.
  A final requirement for such a successful program is the investment of the program director in volunteer growth and learning. Through not only the course but also informal training of volunteers, our program director invested significant time in preparing volunteers with both materials and the knowledge to effectively present these programs. Consistent feedback before, during, and after presentations further allowed volunteers to continue growing as communicators and science educators. This investment guaranteed the preparedness and education of volunteers.
2. **Effective presentations: Pedagogical approach focused on one topic.** Presentations must be handpicked to meet each event’s educational need, while retaining creativity and excitement. Choosing one topic and demonstrating it via several engaging activities are key. Even if the demonstration could teach many interesting points, only one lesson is selected. Since audiences vary widely with regard to age and education level, the director and community partner must first decide who the target audience will be and tailor the presentation accordingly. By meeting face-to-face prior to establishing a new partnership between community partner and DCO, the program coordinator is able to clearly identify the goal of the outreach from the community partner.
  This one-concept approach was developed over the program director’s 23 years of teaching experience and doctoral work studying effective teaching styles in the field of chemistry education. Through this research, the concept of a pedagogical approach using multiple different modalities produced an efficient and engaging teaching method, conveyed to the volunteers at required practice sessions and in the Chemistry Learning Service Learning course.
3. **Investment and education in volunteers: Reflection and refining presentations.** The process of self-reflecting and self-evaluating performances is critical to the growth and evolution of presenters and their presentations; by sharing their experiences and insights, presenters develop their own intuition for what works in a presentation [12]. DCO requires presenters to reflect on their own performance and effectiveness of their communication. Ideas for improvement are often discussed with copresenters and the director.
  Students in this course received formal training on presenting for DCO through 3 separate modalities all centered around feedback: (a) peer feedback, (b) self-reflection, and (c) professor feedback. Throughout the course, students were required to present an outreach performance for the entire class while being recorded by videotape. The student received feedback from both the professor as well as the entire class immediately following the presentation. The student would then watch this video and self-evaluate, a recognized strategy for improving teachers’ communication and effectiveness [13,14]. Lastly, following each outreach event, feedback from copresenters, the coordinator, and the community partner were elicited and discussed. Through this repeated cycle of formal and informal reflection, student presenters flourished as effective educators.
4. **Sustained financial and administrative support.** The main expenditure for any outreach program includes consumable materials, permanent equipment, and travel. All DCO events were completely free for the community partner. Initially, the program was funded through intrainstitutional funds, but as the program grew and the intrainstitutional funding became more limited, external funding was required. Fortunately, local community organizations partnered with our director to identify local sources of long-term, consistent funding. Acquiring dependable funding is critical to ensuring on-going success of DCO and other similar programs.

## Conclusions

DCO is a fun-filled volunteer outreach program that has had far-reaching impact across NC and the greater Eastern seaboard. We have described a cadre of experiments for all ages as well as keys to success. These experiments span the range of both cost and number of materials required; however, all are adaptable to venues varying from university laboratories, local museums, science fairs, and grade-school classrooms. At our institute, 7 separate departments or institutes developed and presented outreach alongside DCO. Utilizing a pedagogical approach to outreach, engaging community partners directly, and providing training for our volunteers, DCO built a successful and far-reaching program adaptable across the sciences.

A pedagogical approach to chemistry outreach moves beyond classroom textbooks and utilizes active demonstrations to mentally engage the audience in the demonstrations presented. This approach requires the presenter to first, master the concepts being taught; second, elicit the background of the audience and knowledge base; and finally, deliver the presentation in an effective and engaging manner [12]. Research into beset practices in informal science learning are few and far between [8], and certainly programs like DCO allow for opportunities to further research in informal science learning. Current guidelines from the National Academies of Sciences, Engineering, and Math provide robust recommendations for effective communication [8].

Science outreach programs such as DCO not only benefit the surrounding community but also help volunteers learn improved science communication and self-confidence, often inspiring a love of teaching. The program’s design creates a higher level of flexibility and versatility that reaches a wide range of audiences, spreading scientific engagement and improving science literacy in the surrounding community. While time and funding are certainly challenges to establishing such a program, the value to both community members and volunteers alike is indisputable, and the joy of our presenters, learners, and community partners is truly contagious.

## Supporting information

S1 Table List of DCO events 2005–2019. DCO, Duke Chemistry Outreach.
(XLSX)

S2 Table Demonstrations with links to the full description of the experiments, including the science, set-up, experiment, and takeaways for our experiments. Of note, Bubble-ology has been performed in many museum settings to great delight, and “The Case of Missing Bunny” and “Chemistry and Our Health” are 2 series of 6 experiments adaptable in a wide array of settings including Alumni Weekend events for kids, science days at elementary schools, and After School Science Club.
(XLSX)

S3 Table Reading and course description for Chemistry Service Learning course. (XLSX)

S1 Text Syllabus for Duke Chemistry Service Learning Course. (PDF)

## Abbreviations

DCO: Duke Chemistry Outreach
IRB: Institutional Review Board
STEM: science, engineering, technology, and math
UI: universal indicator
