# Supplementary material for: A pedagogical approach to science outreach
Source: PLoS Biol. 2020 Apr 16;18(4):e3000650. doi: 10.1371/journal.pbio.3000650 (PMC7188294; doi:10.1371/journal.pbio.3000650)
Supplement: S1 Text — (PDF) [file pbio.3000650.s004.pdf]

Chemistry Outreach: Sharing Chemistry with the Community

COURSE OVERVIEW:

Chemistry 109 is about the opportunity for you, the student, to share your interest in and enthusiasm for science, specifically chemistry, with the general populace residing within the communities surrounding Duke University. The primary focus of the chemistry outreach program is on K-12 level students, however, presentations for tertiary level students and for the general public are also included in the program.

Chemistry109 is designed to prepare you for staging chemistry outreach presentations. You will learn to perform numerous chemical demonstrations. There are several knowledge skills associated with learning a chemical demonstration that will be addressed in this course and you will be expected to develop: 1) the selection of a demonstration that is appropriate for the audience and the venue, 2) an in-depth understanding of the relevant chemistry, 3) practical knowledge of the chemicals used and produced with an emphasis on safe handling and disposal, 4) the mechanics of performing the demonstration, 5) age-appropriate pedagogical strategies that promote understanding of the chemical concepts, and 6) evaluative techniques to assess the effectiveness of the presentation both during and afterwards.

Chemistry 109 includes a rigorous service-learning experience. You are required to complete a minimum of 20 hours service in the community staging live chemical demonstration programs. The majority of the venues will be primary, secondary, and tertiary educational institutions. Other venues in the past have included public libraries and museums, and the North Carolina State Fair. You will develop and demonstrate skill in reflecting critically on issues that arise in your service learning experiences, including ethical issues, public policy questions as they relate to the teaching of science, and issues surrounding civic responsibility and what it means to be a citizen.

Reflection is a major component of Chemistry 109. Recently the President of the United States has mandated that the Nation's schools place a greater emphasis on science and mathematics education citing a decline in the performance by American children in science and math; the importance of having a scientifically literate society; and the need for future scientists if we are to solve the problems facing all of us as citizens of planet Earth. Concern for the decline in the number of U.S. born citizens who choose to pursue a career in science as well as the under-representation of certain groups of people in scientific fields has also been voiced. Research suggests that intervention early in one's educational experiences can have a positive influence on one's attitudes about science and about one's abilities to do science. Research also suggests that the presence of appropriate role models of individuals who do science can also have a positive affect on one's view of his or herself as a potential scientist. With these points in mind, what role can chemistry outreach presentations play in fostering these desired attitudes of science and of one's personal perspective of doing science? And what possible role can you the presenter play in fostering positive attitudes? In Chemistry 109 we will explore and you will reflect upon these issues and others that arise during the course through readings, discussions, questions, and actual experiences staging outreach presentations.

CLASS SESSIONS:

Beginning with the second week the format for each class session will be as follows:

- Tuesdays
    - Class discussion of assigned journal articles; two students will be selected to serve as the discussants for each session.
    - In-class individual presentations of chemical demonstrations; two students per session will be selected to perform an individual demonstration; the other students in the class will provide immediate feedback/critique of the demonstration performances. A video record of the demonstration presentations will be made available on Blackboard for you to view in private and to write a self-critique. Self-critiques will be due the week following your presentation.

- In-class group presentations begin March 2; groups of 4 students will present a series of demonstrations that address a theme (one or more chemical concepts such as the "like dissolves like" rule); the other students in the class will provide immediate feedback/critique of the presentation. A video record of the presentations will be made available on Blackboard for each group member to view and to write a critique. Critiques will be due the week following your presentation.
- Note: there will **not** be any demonstration presentations Week 2; only a discussion of the readings.

- Thursdays
  - Post-outreach reflections
  - General discussion and planning for up-coming events
  - In-class practice.

OUT OF CLASS PRACTICE SESSIONS:

The out of class practice sessions provide the opportunity for you to select, try out, and practice a number of chemical demonstrations. Practice allows you to develop the confidence and skills associated with staging an effective chemical demonstration. The following are my expectations of what is to occur during each practice session:

- Attend a minimum of one practice session per week for at least one hour (consider this practice as part of your *homework*.)
- As this is a laboratory setting, you are to be properly dressed, wear goggles, and other personal protection equipment as dictated by the nature of the demonstration to be performed.
- Select a demonstration and discuss your selection with me. In the discussion you should be able to describe:
  - The demonstration and associated chemistry—What concepts are being addressed and how does the demonstration address the concepts?
  - Materials and equipment needed to perform the demonstration.
  - Safety issues including MSDS information for materials used and how you will handle any waste generated.
- Gather all necessary materials and prepare required solutions for the demonstration.
- Perform the demonstration repeatedly until you are confident with the mechanics of performing the demonstration and are familiar with the outcomes.
- Keep a record of each practice session in your journal (see below).
- Keep the lab clean and orderly.
- Safely dispose of any chemical waste generated.
- AT NO TIME SHOULD ANYONE BE IN THE LAB PERFORMING DEMONSTRATIONS ALONE.
  - After you have become competent in performing the demonstration you may come into the lab to practice WITH ANOTHER STUDENT ENROLLED IN THE CLASS without supervision. However, I need to be made aware that you will be doing so prior to actually coming in for practice. Students not enrolled in the course are not to be in the lab at any time.
  - NO NEW DEMONSTRATIONS ARE TO BE PERFORMED WITHOUT MY IMMEDIATE AND DIRECT SUPERVISION OR THAT OF THE TA.

SERVICE-LEARNING:

The service-learning component of this course allows you to put into practice what you have learned in the class and provides you with the opportunity to share your enthusiasm for chemistry with the general populace in a variety of venues. The format of each event depends upon the goals of the program and/or the specifics outlined by the requesting party. For example, middle school instructors have requested programs in the past that focused on the nature of science, physical and chemical changes, exo- and endothermic processes, and safety, among other concepts. A list of venues from past outreach

performances can be found at the end of this syllabus. Detailed descriptions of the various venues will be given on the first day of class and again as they arise during the term. You are expected to do a minimum of 20 hours of actual chemistry outreach presentations. The time spent in preparation, travel, and post-presentation reflection is considered as part of this 20-hour commitment. You do not need to seek out venues to complete the service-learning component. I will work directly with the requesting parties in planning and staging of the outreach presentations. However, if you wish to do so, you may contact and work directly with a school or other venue in staging an outreach presentation after discussing your plans and obtaining approval from me to go ahead. Arranging time to participate in the outreach events around the rest of your academic schedule can prove to be the biggest challenge for you in this course. Often requests from schools are for performances during their regular school day and some events are staged outside of the immediate area around Duke. However, there are several evening and weekend events that should provide ample opportunities to complete the 20-hour requirement. Transportation to and from the venues will be arranged as needed. My expectations are as follows:

- Be committed
    - You can be depended upon to show up and give your best in all aspects of the chemistry outreach event.
- Be prepared
    - You are prepared to present your demonstration in a skilled and confident manner.
    - You have prepared all necessary materials for the demonstration and ensured everything needed has been brought to the venue.
    - You are knowledgeable of the chemistry relevant to the demonstration to be performed.
- Be on time
- Be positive
    - You are a positive role model.
- Be safe
    - You portray safe chemical practices as you perform the demonstration.
    - You are aware of your personal safety and that of the audience at all times.
    - You dispose of all waste following appropriate procedures.
- Be friendly
- Be clean
    - Leave the venue as clean, if not cleaner, than when you arrived.

COURSE REQUIREMENTS:

- Attendance
    - The class will meet two days per week for 75 minutes. Regular attendance is very important and one of the determinants of your final grade. If you cannot attend, please try to let me know **in writing** beforehand.
    - You will also be expected to come to the lab weekly on your own time in order to practice chemical demonstrations. A minimum of one hour per week is expected but you may opt to spend more time, especially prior to the staging of an outreach presentation. Scheduling of times will be based on convenience for both you and the instructor or TA.
    - You will only be permitted three absences per semester. After this point, your grade will be affected.
    - **If you miss a presentation in class, you must watch the video online and write the critique, turning it in the following class period. You must also submit your reflections on the assigned readings.**

- Participation
    - Your active participation is essential to the success of this class. Participation involves not only contributing your ideas, but also actively listening to other class members. There are at least five facets of participation.
        - Participation in class discussions of assigned journal articles.
        - Participation by performing in-class demonstrations.

        - Participation in critiques of demonstrations performed by other class members in class and your self-critiques.
        - Participation in the planning, preparation, and presentation of outreach programs.
        - Participation in the post-presentation evaluation discussions.

- Reading
    - There is no text for this course.
    - You will be provided with copies of articles from recognized journals dealing with a variety of topics applicable to this course. You are to carefully read each article when assigned, record your thoughts/reflections about what you read in your journal, and prepare for class discussion.

- Reflections
    - You will be expected to maintain a journal using the Blackboard Wiki Tool to keep track of your Service Learning experiences, your weekly practice hour, and the assigned journal articles.
        - *Record of Outreach Performances*: After each chemical outreach presentation you are to reflect upon what took place recording your thoughts as to what went well, what needs improvement, and ways the presentation might be improved.
        - *Journal Articles*: You will be assigned several journal articles to read throughout the term. You are to record your thoughts as you read each article and your overall reflections after reading the entire paper. This is not to be a summary of the article. It is a record of the thoughts, ideas, reflections, and questions that arose as a result of reading the article.
        - *My Space:* In this section you have space to record any other thoughts, ideas, reflections, and questions that arise during the course which are not specifically addressed in the other three sections of your journal. Specific questions you have that you would like for me to respond to, please leave about one-quarter of a page blank after each question.
    - You will be expected to share some of your experiences from outreach in class the following week.

- Service-learning
    - You are required to do a minimum of 20 hours of actual chemistry outreach presentations over the course of the term. Preparation, travel, and post-presentation reflection are considered as part of this time commitment. There are a variety of venues and formats available to you in order for you to meet this requirement (see list at the end of the syllabus).
    - Please do not include the hours you spent traveling when you fill out the form to record your service learning for us. In your journal, however, you will record this, and we will take this into account.

GRADING AND EVALUATION:

In determining your final grade, emphasis will be placed on attendance, class participation, your understanding of the readings as demonstrated in discussions, your in-class demonstration presentations and self-critiques, your service-learning experience, and your journal. My expectations are that you will arrive to class on time and that you will meet each of the class requirements. You are responsible for your "academic self." If at any time during the term you are concerned about your grade, please speak with me.

Your final grade will be calculated as follows:

**Item**	**Percentage of Total**
Attendance and participation	25
In-class demonstrations and self-critiques	25
Service-learning experience	25
Journal	25

**Letter Grade**	**Basis**
**A+**	Student has met all requirements for the course and has excelled in all aspects
**A**	Student has met all requirements for the course and has excelled in most aspects
**A-/B+**	Student has met all requirements for the course and has excelled in some aspects
**B**	Student has met all requirements for the course
**B-/C+**	Student has met the majority of the requirements for the course but is lacking in some aspects
**C**	Student has not met all requirements for the course but the quality of what has been met meets criteria for completion
**D**	Student has failed to meet the majority of the requirements for the course
**F**	Student has failed to meet the majority of the requirements for the course and the quality of what has been met does not meet criteria for completion

Note: Grades of A+, A-, B+, B-, C+ will be determined by the instructor and TA for the course and are not subject for debate.
